# Working Sandwich Generation Women Utilize Strategies within and between Roles to Achieve Role Balance

**DOI:** 10.1371/journal.pone.0157469

**Published:** 2016-06-15

**Authors:** Kiah L. Evans, Jeannine Millsteed, Janet E. Richmond, Marita Falkmer, Torbjorn Falkmer, Sonya J. Girdler

**Affiliations:** 1 Occupational Therapy Program, School of Medical and Health Sciences, Edith Cowan University, Perth, Western Australia, Australia; 2 School of Occupational Therapy and Social Work, Curtin University, Perth, Western Australia, Australia; University of Tuebingen Medical School, GERMANY

## Abstract

Increasingly, women simultaneously balance the roles of mother, parental carer and worker. However, individual role balance strategies among these working ‘sandwich’ generation women have not been thoroughly explored. Eighteen women combining these three roles were interviewed about their individual role balance strategies. Findings were identified through the framework analysis technique, underpinned by the Model of Juggling Occupations. Achieving and maintaining role balance was explained as a complex process accomplished through a range of strategies. Findings revealed the women used six within-role balance strategies: living with integrity, being the best you can, doing what you love, loving what you do, remembering why and searching for signs of success. The women also described six between-role balance strategies: maintaining health and wellbeing, repressing perfectionism, managing time and energy, releasing responsibility, nurturing social connection and reciprocating. These findings provide a basis for health care providers to understand and potentially support working ‘sandwich’ generation women.

## Introduction

Societal trends, such as delays in childbirth and an aging population, have resulted in an increasing number of women simultaneously balancing the roles of mother and parental carer [1–3]. These women have become known as the ‘sandwich’ generation [4,5], and while their numbers are increasing [1], to date research examining the experience of ‘sandwich’ generation women who are also in the paid workforce has been limited (e.g. [6,7–9]). Some evidence suggests that the combination of these three roles can have detrimental effects, such as time squeeze, negative emotional spill-over and unrealistic expectations [7,8,10–12]. There is also evidence of positive experiences, resilience and coping among working ‘sandwich’ generation women, through sharing skills, energy or resources between roles [7,12,13]. This indicates the presence of protective factors and individual role balance strategies that allow these women to balance their multiple roles and achieve positive outcomes [13]. To date both sandwich generation research and the role balance related literature has focused on the association between personal and environmental factors with role balance related outcomes (e.g. [14,15–17]). However, limited research has examined the individual role balance strategies used specifically by working ‘sandwich’ generation women that might mediate the impact of personal or environmental factors on role balance [7,13].

Personal protective and risk factors have not been investigated extensively for the sandwich generation, but have been well covered in the role balance related research in relation to age, ethnicity, socioeconomic status and religiosity [15,17–23]. Gender differences exist, with working sandwich generation women experiencing more diverse role related challenges, utilizing different individual role balance strategies and having poorer role balance outcomes than men with the same role combination [7,24–26]. There is also evidence of an association between role balance related concepts and numerous psychological variables, such as affect, emotional intelligence, locus of control, personality traits and types, resilience, self-efficacy and stress [14,17,18,22,27–29]. Research also indicates role balance related concepts are linked with an individual’s health and functional independence [20,29]. These personal factors may influence role balance related outcomes through facilitating or hindering the use of individual role balance strategies.

Environmental protective and risk factors explored in the sandwich generation and role balance related literature include those stemming from the family, work and societal environments. Family factors found to impact on role balance related concepts include perceived appreciation, relationship quality, caring duration, intensity of caring tasks, diagnosis of care recipient, living arrangements and practical or emotional support received from partners, children, parents and siblings [4,6–8,10,15,16,22,23,30–36]. Work factors associated with role balance related concepts include support from supervisors or co-workers, job demands, work arrangements (such as number of hours, schedules, flexibility and telework) and motivation for employment [15–18,27,37–46]. Collectively, work factors are the most extensively studied variables in the role balance related literature, being the focus of numerous systematic and meta-analytical reviews. Documented societal factors impacting on role balance related concepts include legislation, gender socialization and availability of informal and formal community support services [6,22,36,39]. These environmental factors may be associated with role balance related outcomes due to their influence on the uptake of individual role balance strategies.

Individual role balance strategies identified within the ‘sandwich’ generation and carer literature include psychological approaches, self-management techniques, engaging social supports and participating in self-care activities. Psychological approaches include decreasing expectations of self, optimistic thinking, meaning making, normalizing experiences and practicing gratitude [6–9,22]. Self-management techniques are being organized [6,7,9], exerting control over role participation [7,9,33], prioritizing needs [7,33] and mindfulness [8,9,22]. Engaging social supports differ according to the availability of supports within the environment, as it involves reaching out to request, negotiate or accept the required level of support. This support is obtained through sharing, delegating or outsourcing role demands [6,9,34,36] and communicating within relationships [8,9]. Participating in self-care activities include making and protecting time to be alone, pursuing leisure and engaging in healthy behaviors [6,8,47]. Although three of these studies directly address individual role balance strategies among ‘sandwich’ generation women, one study took a didactic approach to work and family related strategies [7,33] and the other two did not specifically examine their relationship to the worker role [6,9]. The remaining studies focus on challenges [8] or related populations [22,34,36]. The most comprehensive findings stem from Neal and Hammer’s [7] focus groups with dual earner ‘sandwich’ generation couples. Their model included six types of individual role balance strategies, where individuals either decrease demands or increase resources through behavioral, emotional or cognitive approaches. A significant gender difference was noted, where wives were more likely to restrict social or personal time and husbands were more likely to protect time for important activities.

Individual role balance strategies are described to a greater extent among other populations within the occupational therapy literature, driven by the discipline’s focus on participation in meaningful activities and roles [48]. Documented strategies include balancing time and energy resources [18,49–56], balancing individual and collective needs [35,49,53–57] and balancing demands of multiple activities or roles [18,29,49–51,53–60]. Individual strategies to balance the demands of multiple activities and roles have been shown to include participating in a diverse range of activities to meet a variety of needs, with a particular focus on activities aimed at resting, maintaining health, nurturing relationships, creating a positive identity, and pursuing rewards or stimulation [18,35,49,50,52–57].

A holistic understanding of individual role balance strategies among working ‘sandwich’ generation women is essential [4,6,8,33], as there is an opportunity for health care to provide early intervention to assist working ‘sandwich’ generation women to exert control over their circumstances when faced with personal and environmental risk factors. Although the occupational therapy literature provides a strong conceptual foundation for exploring individual role balance strategies, to date no research has drawn on this foundation to explore the experiences of working ‘sandwich’ generation women. Hence, a gap exists in the current research literature regarding the range of individual role balance strategies working ‘sandwich’ generation women utilize to manage their multiple roles.

In addressing this paucity of research, the aim of this study was to obtain an initial understanding of how working ‘sandwich’ generation women achieve and maintain role balance, using the Model of Juggling Occupations [12] as a conceptual framework. The Model of Juggling Occupations explores the concept of role balance in a holistic manner, and focuses on experiences both within and between roles [12,61]. Within-role balance is explored in relation to activity participation, values, interests, perceived competence and habits within each role [12,62]. Between-role balance is explored in relation to the conflicting and enriching interactions between roles [12,63,64]. Drawing on this model, findings from this study will provide insights into the within and between individual role balance strategies employed by working ‘sandwich’ generation women, and will be important in informing future health care provision to support these women to balance their multiple roles.

## Materials and Methods

This study was underpinned by a qualitative case study methodology [65], involving semi-structured interviews with eighteen working women who had dual caring responsibilities for their children and members of the parental generation. Interviews were selected as the most appropriate data collection methods as they enabled an in-depth and personal understanding [66] of the strategies involved in achieving and maintaining role balance. The Edith Cowan University Human Research Ethics Committee approved this study (approval number 08–110). The research was conducted according to the principles expressed in the Declaration of Helsinki. Informed written consent was obtained from the participants. All procedures were piloted prior to commencing data collection [12].

### Sample

Given the gender differences in role related challenges, individual role balance strategies and role balance outcomes experienced by members of the working sandwich generation [7,24–26], it was deemed appropriate to collect data from participants of a single gender. Subsequently, the decision was made to focus exclusively on women during this study, as they faced the greatest risk of poor role related experiences and outcomes [7,24–26]. A convenience sampling approach was chosen as an appropriate approach to allow an in-depth exploration to occur with a homogenous sample of working sandwich generation women [66,67]. The study was advertised extensively through a range of community organizations, businesses and personal contacts, and all women who met the inclusion and exclusion criteria were invited to participate in the study as they volunteered. This was considered the most appropriate sampling approach to recruit the maximum possible number of working women (working a minimum 12 hours per week) who were simultaneously providing care to at least one child under 18 years old and a community dwelling parent or parent-in-law.

The eighteen women who participated in this study were partnered and their age ranged from 32–55 years old (median = 44 years old). The women had a relatively high level of education, with all but four women holding a tertiary qualification (nine women held a vocational certificate or diploma and five women held a university degree). Their income was above average, with a median individual income of $AUD31,200–41,599 (range = $AUD7,800–259,999, where the median income of Western Australian women was $AUD38,552 per year [68]) and a median family income of $AUD104,000–129,999 (range = $AUD26,000–260,000+, where the Western Australian metropolitan average household income was $75,898 per year [69]). Similarly, the women tended to live in suburbs ranked highly on the Index of Relative Socio-economic Advantage and Disadvantage (median = 9^th^ decile, range = 2^nd^– 10^th^ [70]) and almost all lived in a home that they owned either outright (n = 6) or with a mortgage (n = 11) within a metropolitan region.

In terms of their role characteristics, as mothers the women typically had two children (range = 1–4) with a median age of 13 years old (range = 0–27 years). A total of ten women had at least one child with a chronic health issue or disability. As parental carers, all but one woman cared for their mother and approximately half also cared for at least one other member of the parental generation (median = 1 parental care recipients, range = 1–4). Of these, 13 women considered themselves to be primary carers and six women co-resided with their parental care recipient. All women provided their parental care recipient with emotional support and assistance with instrumental activities of daily living, whilst ten women also provided assistance with activities of daily living. Twelve women had been a parental carer for at least two years, with the remaining six women providing care to the parental generation for between six months and two years. As workers, all but three women were employed on a part-time basis, with a median of 24 total hours worked per week including unpaid overtime (range = 12–50 hours). Four women worked exclusively from a home office, whilst three women alternated between a home office and an external workplace.

### Data Collection

The Model of Juggling Occupations informed the development of a semi-structured interview guide aimed at exploring the individual role balance strategies utilized by working ‘sandwich’ generation women [12]. Demographic details were recorded, including marital status, age, education, income, place of residence (as an indicator of socio-economic standing) and home ownership status. Role related information was also collected for the roles of mother (age and health status of each child), parental carer (relationship of each care recipients, primary carer status, living arrangements, type of assistance provided and role duration) and worker (total work hours, including unpaid overtime or second jobs, and work location). In addition to open-ended questions on within-role balance, between-role balance and overall role balance [12], the women were asked to describe the factors that either help or hinder achieving role balance, characteristics of a day where they felt high and low levels of role balance, and what role balance advice they would give to other working ‘sandwich’ generation women (S1 File).

The women reviewed a brief outline of the study and provided written informed consent to participate [66], prior to attending a face-to-face interview in 2010 with the first author at a location of their choosing. The first author is a registered occupational therapist experienced in interviewing working age women. Interviews took an average of one and a half hours, although this varied from just over one hour to three hours, and were digitally recorded with permission. Confidentiality was maintained through assigning pseudonyms and removing identifying information during the transcription process and all field notes were prepared using the pseudonym [66]. A written vignette summarizing the facilitators and barriers to role balance was prepared for each participant. Sixteen women reviewed their written vignette to confirm the accuracy of emerging findings [66,71,72], with the remaining two women unable to participate in the follow-up meeting because of their competing role demands.

### Data Analysis

Framework analysis technique [73] was utilized by the first author to analyze the interview data, with the assistance of QSR NVivo 10 software. First, familiarity with the data occurred through listening to the audiotapes, along with reading the transcripts and vignettes multiple times. Notes on emerging themes were recorded during this period of immersion. The second step involved identifying a thematic framework, which included key concepts from the Model of Juggling Occupations [12], refined based on findings from a pilot study [12] and additional key literature [62–64]. The third step involved indexing the data against this thematic framework. The fourth step involved charting the summarized findings in a matrix of participants and emerging themes. The fifth step mapped and interpreted the data holistically to answer the research question, as a result of comparing, contrasting and combining the findings from earlier stages. This allowed the range of role balance strategies utilized by working ‘sandwich’ generation women to be described in detail, associations between these experiences to be explored and explained, and a taxonomy of role balance strategies to be proposed [73]. Once the taxonomy of role balance strategies had been refined, the first author read the vignettes to identify the types of individual role balance strategies utilized by each woman. The vignettes were reviewed by another author to enhance the trustworthiness of the findings, with this process confirming the individual role balance strategies described in this article accurately reflect the participants experiences [66,72].

## Results

**Fig 1:**
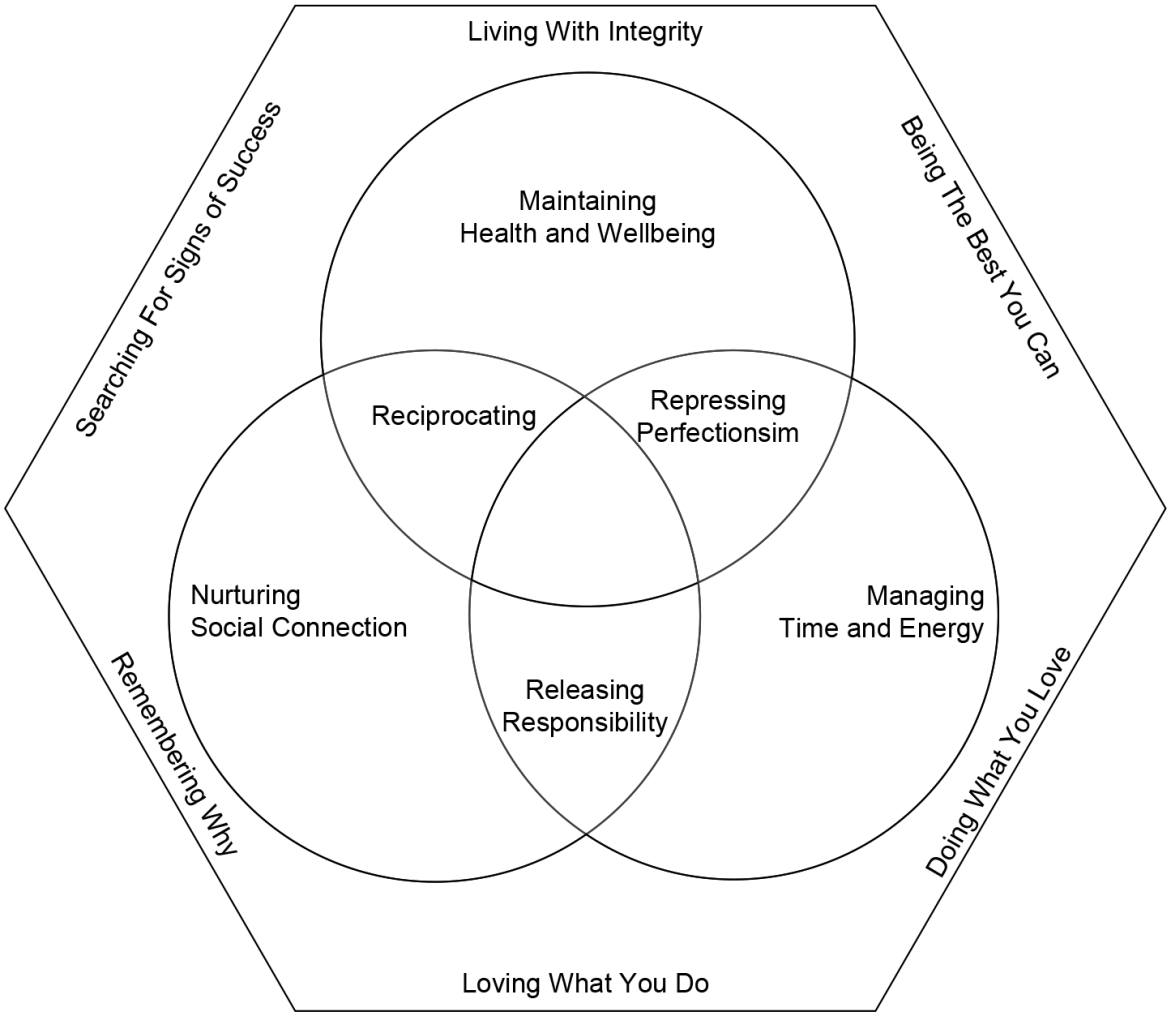
Within-role (hexagon) and between-role (Venn diagram) balance strategies

**Fig 2:**
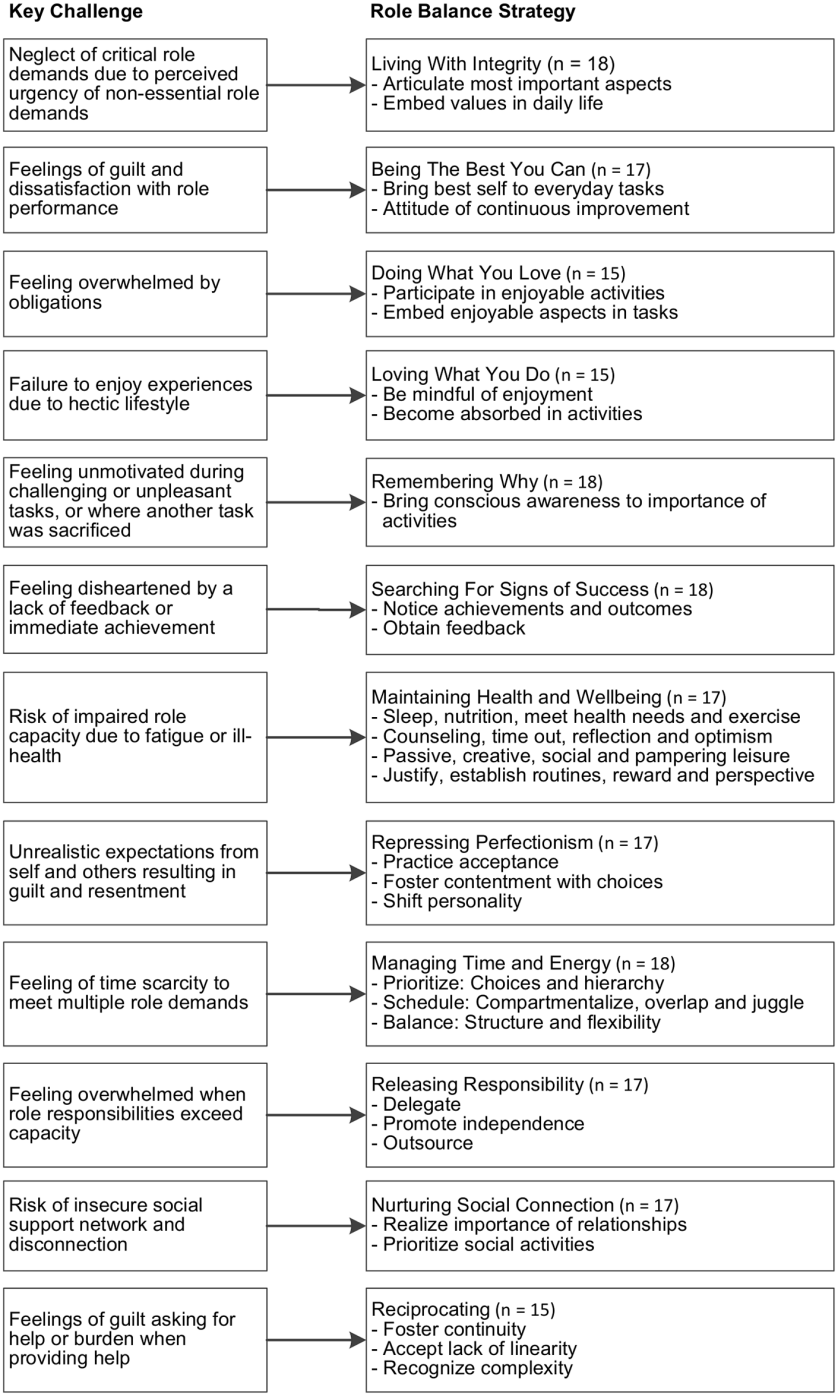
Key challenges faced by working ‘sandwich’ generation women and associated role balance strategies

The women in this study felt passionate that seeking to achieve and maintain role balance was a central life goal, illustrated through “If you can conquer this role balance, you’re the best…. It’s hard, but when you find the structure to it, it changes you. It makes you feel good about yourself. It really does.” Implementing role balance strategies was described as hard work and as not “happening overnight.” Achieving role balance was seen as a long-term goal, the result of a lifetime of consistent effort, maintained by a constant “juggling act.” Twelve main strategies utilized to achieve and maintain role balance within and between roles were identified (Fig 1) to allow the women to address a range of challenges (Fig 2).

### Within-Role Balance Strategies

The women in the current study consistently described six within-role balance strategies, which enabled them to effectively arrange their habits and routines in line with their motivational factors. Quotes illustrating each strategy for the roles of mother, parental carer and worker are provided in Table 1. These strategies were: living with integrity, being the best you can, doing what you love, loving what you do, remembering why and searching for signs of success.

**Table 1:** Example Quotes to Illustrate each Within-role Balance Strategy for the Mother, Parental Carer and Worker Roles.

	Mother	Parental Carer	Worker
Living with Integrity	“If he wants to read a book, or if he wants to play a game, then I’ll tend to just drop what I'm doing.”	“I don’t see it as a burden. I see it as just part of life…. It fits in with our values.”	“I make sure I do my work from A to Z before I leave…. I make sure that I’ve left everybody really satisfied.”
Being the Best You Can	“It’s important for me when they open their lunch box to know… ‘Mum’s really thought about us.’”	“I’ve done my best to make decisions that I thought were in her best interests.”	“I really do feel when I go to work… I give everybody my all.”
Doing What You Love	“Time spent together in a positive way…. That the time is encouraging and uplifting and worthwhile.”	“A lovely opportunity for memory building…. I think that’s a really lovely thing to build into the family.”	“Every time I'm working I feel stimulated…. I get excited… I feel that I work just to remain stimulated.”
Loving What You Do	“Cherish the time… focus on those joys.”	“We’ve got time together…. Every day is precious.”	“It makes you feel great… it just gives you such a buzz.”
Remembering Why	“Being a mother is the most important role…, because at the end of the day, these are going to be the people who become parents in the future.”	“I feel that it’s really important for me to give mum the support that not only that I feel that she needs, but she feels that she needs.”	“If I was going to be away from the boys… it would be for something that was worthwhile…. I don’t just want to be wasting my time away from them.”
Searching for Signs of Success			
Direct Feedback	“My daughter will quite often say ‘I'm so lucky that I’ve got a mum like you and thanks for everything you’ve done.’”	“Most times my mum will say ‘Thank you for doing that, it’s so nice to have you here, I really do appreciate it.’”	“I had my performance appraisal and [my manager] rated my work as ‘above and beyond on a regular basis.’”
Feedback from Outcomes	“The kids are all dressed and fed… or even getting in the pram and going for a walk is an achievement.”	“Peace that I’ve done my duty…. That I’ve given what I can give, in the situation that I have.”	“Sense of satisfaction of knowing that you’re able to do highly complex things.”
Feedback from Observation	“Your children are a product of you… they’re respectful, they’re polite, they’re good people, they know right from wrong, they’re caring, and they support each other as well. So that does heaps to help my confidence.”	“You can just hear it in mum’s voice. She wants to talk away, she just doesn’t want you to go…. And it’s just that feeling that … they appreciate you just being there. So that makes me feel really, really good.”	“I see clients grow, it’s just so beautiful to see…. And just to be that provider of information and to have that continuity of care over that timeframe is so privileged.”
Feedback from Others	“When they do well, no matter how old they are… and it’s not just about awards, it’s the recognition. It makes me feel ‘Well, I have achieved something. I’ve helped them get there.’”	“My aunty will say to me, ‘Mum said that you did this’…. And that’s when I know that mum’s had some enjoyment from it. But it’s not necessarily from mum, it’s from a third party.”	“Quite often other staff will comment ‘It’s really good how you handled that’…. Other people are obviously taking account of what you’re actually doing, above and beyond what you normally do.”

#### Living with integrity–“It fits in with our values”

Living with integrity refers to the women’s strategy of participating in activities and roles in a way that was congruent with their values, and all women practiced this strategy. Being true to their values ensured that they did not neglect critical role demands because of the perceived urgency of non-essential role demands. All women articulated the most important aspects of each key role, and within-role balance was enhanced when their values were frequently embedded in their daily life. The women strived for “a little bit more quality” in these important aspects. Being able to integrate their values into daily life allowed the women to experience a sense of peace: “I don’t see [caring for family members] as a burden. I see it as just part of life and part of our responsibilities… It’s that it fits in with our values.” Congruence between roles and values led to feelings of within-role balance.

#### Being the best you can–“I give my all”

Being the best you can refers to the women’s strategy of striving to be “a very capable woman”, with high levels of competence and confidence within each role. This strategy was utilized by 17 women to reduce feelings of guilt and dissatisfaction in relation to role performance. The women enacted this strategy by consciously bringing their “best selves” to everyday tasks. Even when a role was challenging or they felt unappreciated, the women subscribed to the mantra “I try to do the best that I can”, allowing them to “feel settled” in themselves regardless of the outcome. The women described having an attitude of continuous improvement, with mistakes providing an opportunity for personal growth: “Just knowing that I’ll do my absolute best to be the best I can be. I’ll make mistakes, I know I will… but I’ll learn from them and make it better and better, hopefully.” Striving to do their best enhanced the women’s competence, confidence and within-role balance.

#### Doing what you love–“I actually quite like it”

Doing what you love refers to the women’s strategies of creating opportunities to engage in enjoyable activities and embedding fun into roles. This strategy was utilized by 15 women to avoid “just being a slave of ‘this is what I’ve got to do.’” The women felt it was important to frequently create opportunities for enjoyable activities, and felt “thrilled and happy” at accommodating even brief periods of “15–20 minutes” in their busy schedule. Embedding enjoyable aspects into roles included displaying a playful attitude, socializing or overlapping tasks during daily activities. An example was “Until recently I couldn’t stand ironing, but now I actually quite like it because I put [a DVD] on the computer and I get into a zone and I iron and it’s kind of cool.” By engaging in activities they loved, the women enhanced their experience of joy and within-role balance.

#### Loving what you do—Notice “the fun of everything”

Loving what you do refers to the women’s strategy of deliberately noticing, and becoming absorbed in, the enjoyable aspects of the present moment. This strategy prevented 15 women from missing enjoyable role related experiences “because [they] happen so fast.” The women focused on “the fun of everything”, even though they had to consciously “stop and think about it”, take a mental “snap shot” and “try and remember those things.” They subsequently felt “it’s so nice to be absorbed” in these enjoyable aspects, and this state of absorption was “a real coping strategy” because it allowed them to not “think about anything else” and “switch off” from their daily responsibilities. For the women in this study, actively focusing on the enjoyable aspects of their activities and roles enhanced positive emotions, role engagement and within-role balance.

#### Remembering why—“I will feel the benefits later on”

Remembering why refers to the women’s strategy of bringing conscious awareness to the importance of their current activity or role. This strategy was utilized to some extent by all women when they felt conflicted or when the tasks were challenging or unpleasant. Through this strategy they recognized that “even though I feel as if I’m struggling now, I will feel the benefits later on.” Commonly cited reasons for role participation were opportunities to meet their “duty”, minimize the “guilt factor”, “make a difference”, “feel more validated” and “contribute financially.” In remembering why they prioritized an activity, the women were able to buffer the impact of an absence of enjoyment or success and maintain their within-role balance.

#### Searching for signs of success–“The sacrifices have paid off”

Searching for signs of success refers to the women’s strategy of looking for indicators of success in their roles when it was not immediately apparent that they had fulfilled their roles. This strategy was utilized by all women to counteract feeling disheartened by a lack of direct feedback, as they typically needed to actively search for signs of role success that were not obvious. Signs of role success were not always immediate, as noted with “I don’t think you get to experience [the feeling that you have done the right thing with your children] until they start to grow up, and you actually see them starting to make their own decisions and choices. Then you know what you did maybe three years ago was valuable.” This strategy fostered role balance when they stopped and noticed they “have managed to balance it. Perhaps some of the sacrifices that you’ve made … have been of benefit and have paid off.” When signs of role success were not obvious, the women actively searched for these indicators through noticing the outcomes of their actions and observing or requesting feedback from others.

Feedback from outcomes involved celebrating their achievements, from achieving long term goals through to finishing repetitive tasks. The women felt particularly proud when these achievements required “pure endurance, patience and being caring.” Celebrating these achievements did not need to be elaborate or formalized, with one woman admitting she “would do a dance” on certain successes. The quote “At the end of the night after the dishes are done, you have that sense of another day is done. That feeling of ‘I won, I got through another day’” highlighted the powerful impact that within-role balance had on the women’s sense of feeling successful across the day.

Feedback from observing others for signs of successful role performance involved watching for non-verbal cues and actions in their loved ones. The women felt strongly that even “those small gestures do mean a lot”, and though not always immediately apparent for the women in this study they were important indications of success. These observations enhanced their sense of pride, appreciation and within-role balance.

Feedback from others was obtained by conversing with their wider social networks, and provided the women with evidence of role success. This type of feedback was most helpful in maintaining feelings of success when they felt they were not appreciated by those they cared for: “[My Mum] is not a person who actively says thank you.” This indirect feedback from others was a source of gratitude and within-role balance for these women.

### Between-Role Balance Strategies

The working ‘sandwich’ generation women in this study described six between-role balance strategies that acted to minimize between-role conflict and maximize between-role enrichment. These strategies were: maintaining health and wellbeing, repressing perfectionism, managing time and energy, releasing responsibility, nurturing social connection and reciprocating.

#### Maintaining health and wellbeing–“Finding time for yourself”

Maintaining health and wellbeing refers to the women’s strategy of “finding time for yourself, even when it seems impossible.” It was the most advocated between-role balance strategy among all women, although only 17 women reported currently attending to their health or wellbeing. This strategy preserved the women’s capacity for role participation, and was achieved through actively attending to their physical health, mental health and wellbeing.

Preserving their capacity for role participation was perceived as “important in the long run.” One woman explained, “[Working ‘sandwich’ generation women]… are the linchpin for the family and if we don’t look after ourselves then everything else is just going to fall apart.” This was further explained with, “You’re no use to anybody if you haven’t got your own energy on board” and “It empowers you to give more, because you’re giving yourself more.” Another woman declared finding time for herself was “the biggest thing I’ve changed in my life” because it allowed her to recover from a “breakdown.”

Attending to their physical health, mental health and wellbeing needs was achieved through taking the proactive measures described in Table 2 to ensure they had sufficient time to participate in the necessary activities. Physical health was maintained through sleeping, eating nutritiously, meeting health needs and exercising. The comment “I love to keep reasonably fit to keep up my energy levels. It also helps keep a balanced mind. I reflect and plan while exercising” illustrates that exercise contributed to both physical and mental health. Mental health was also maintained through counseling, taking time away from other roles, reflecting and optimistic thinking. Finally, wellbeing was maintained through leisure time spent in passive, creative, social and pampering activities. These stress-relieving opportunities for leisure typified the concept of “time for yourself.” For the women in this study maintaining wellbeing in addition to maintaining physical and mental health was essential to experiencing between-role balance.

**Table 2:** Types and Examples of Activities to Maintain Physical Health, Mental Health and Wellbeing, Along with Common Maintenance Strategies.

Domain	Types of activities and maintenance strategies
Physical health	Adequate sleep was achieved through earlier bed times or combatting accumulated sleep debt on the weekend through “a sleep in” or nap.
	Nutritional needs were met when they allocated time for regular healthy meals, often through overlapping mealtimes with socializing and ensuring healthy ingredients were available.
	Meeting health needs included maintaining currency with health screenings and treatments by attending scheduled appointments with medical and allied health professionals.
	Formal exercise included group fitness classes, competitive sports and training sessions. Informal exercise was more common, and included deliberate walking or running and incidental exercise embedded in daily activities (for example pushing a pram was “a really good cardio-vascular workout”).
Mental health	Counseling was described as “like taking my vitamins… I do have a very fast paced life, I do have a stressful job, all that stuff and I just need it… to unload. And sometimes you just need someone to tell you that it’s okay.”
	Taking time away from other roles was achieved through following advice to “walk away for five minutes just to clear your head”, “close the door or just sit in your room”, retreat to a “tranquil” location or remember to “breathe.”
	Reflecting alone or with others reinforced feeling “I don’t think I'm alone, I think a lot of people are just like me”, facilitated problem solving by articulating barriers and potential solutions, and enabled acceptance of temporary challenges because they believed “this too shall pass, we’ll get through it.”
	Optimistic thinking involved seeing the opportunities and benefits of multiple role occupancy. Seeing the opportunities was exemplified with the literary reference “Pollyanna plays this Glad Game and it’s very good training … to think of your glass as being half full and not half empty” and the comment “aren’t we just lucky.” An example of seeing the benefits, or “that sense of dreaminess about why you do it”, in relation to her domestic responsibilities was “I love to be able to have that place where everyone knows it’s home and everyone comes together.” Other benefits of multiple role occupancy were role modeling positive behaviors, recognizing the value of intergenerational relationships, affording a better lifestyle and contributing to identity.
Wellbeing	Passive leisure activities included watching television, reading and playing computer games, for example “I get my time to me in the evening. I tend to have a good hour or so before bed to play my Nintendo or read my books or chat to my husband or whatever it might be.”
	Creative leisure hobbies comprised sewing for pleasure, playing piano and painting.
	Social leisure interests took place with friends or family, and included watching movies, attending book clubs and other “activities that I enjoy the most—like going to the beach with the family and unwinding.”
	Pampering as a form of leisure included hair appointments, facials, manicures, massages and foot soaks. Pampering activities were described as “my treat”, “it’s one of those things that I feel that I have to really do for me to maintain my sanity” and “it kept me balanced… It kept me being me… It kept me being a good everything.”

Integrating sufficient health and wellbeing maintenance into routines was “easier said than done. It’s hard, but you have to find time.” Whilst the majority of women were successful at integrating these self-care activities into their routines to some extent, the six mothers of pre-school aged children found this substantially more challenging. The women ensured sufficient time through establishing effective routines, coordinating schedules with their partner, anticipating the rewards and managing their perspective. Effective routines involved setting small daily goals of “even just ten minutes”, scheduling morning activities so “When things get a bit hectic, at least I can stop and think ‘Well, it’s okay, you’ve had some me time’” and participating in fixed-time activities where they felt obliged to attend (e.g., “I have to go”). Coordinating schedules with their partner ranged from asking their partner to take care of their children while they attended an appointment to highly structured routines that allowed both partners to attend to their health and wellbeing on a daily basis. Anticipating the rewards was explained by “Sometimes I don’t want to do it and I force myself. Even during it I'm thinking ‘what am I doing here?’ But afterwards, I feel great, and that’s what makes me go again the next time.” Other rewards included achievement and freedom, as described with “once you’ve done it, you’ve ticked it off. Then you can have more time to do those other things that you need to too.” The women managed their perspective by learning to “Become a lot more selfish and not think about everyone else…. It takes a lot of practice, but it becomes easier the more you do it”, remembering that “it's a very small proportion that I'm giving to myself, compared to what [I’m] actually doing in the whole picture”, having the insight and confidence to “say when they needed a break” and verifying that other role responsibilities had been met.

#### Repressing perfectionism–“Not being super human”

Repressing perfectionism as a way of life refers to the women’s strategy of refuting their own, or others, unrealistic expectations to be “super human.” This strategy was utilized by 17 women to combat guilt and resentment, and involved practicing acceptance, fostering contentment with priority choices and a shift in personality.

Practicing acceptance that “everything can’t be perfect” was an important aspect of repressing perfectionism, as they needed to believe, rather than simply know “There’s only 24 hours in the day and you’ve got to work backwards from that.” Once the pressure to be perfect in all roles at all times was removed, the women focused their attention on achieving a threshold level of role performance. It was deemed “hard to maintain balance with, and quality with, a lot of different things” and hence the women accepted that they needed to limit the number of roles they engaged in. Recognizing that “no one usually cares except you” allowed the women to feel at peace with letting go of unrealistic quality standards.

Fostering contentment with priority choices minimized frustration because “You make a decision that this is a priority and you accept that it’s going to impact negatively” on the ability to meet other role demands. In addition, “That sense of happiness comes from being able to give those things up, but feel that it’s the best thing to do” demonstrated how this strategy facilitated contentment and between-role balance.

A “shift in personality” was required to repress perfectionism for many of the women, illustrated with “It is in my character to want to achieve or to be good or excellent or perfect—or close to perfect—in all my roles.” This was achieved through changing patterns of self-talk, such as frequently reciting that “you can only do what you can do” and repeatedly remembering to “try to take ‘should’ out of [your] vocabulary.” The women described having to actively persist with these thought patterns, in order to manage unrealistic expectations, guilt and conflict associated with perfectionism.

#### Managing time and energy–“A framework”

Managing time and energy refers to the women’s strategy of choosing how to utilize their time and energy efficiently and effectively to meet their many role demands. This strategy was utilized by all women to combat feeling overwhelmed by “the pure magnitude of all the things that need to be done.” Managing time and energy was achieved through the related processes of prioritizing, scheduling and implementing a framework that balanced structure and flexibility.

Prioritizing time and energy refers to the women’s decision-making process regarding what need to be done, once they acknowledged they were “trying to do too much”, and it was utilized because “You get to the point where you have to just realize you can’t do everything.” The importance of prioritizing was emphasized when role balance was defined as “priority balance … it’s a balance between this priority versus that priority, and which means most to you.” The women successfully prioritized activities, making priority choices according to their personal hierarchies of role importance.

Priority choices were made through an internal “priority conversation”, where they compared the relative importance of all role demands on a continuum between essential (“what you have to do”) and desirable (“what you want to do”). This also involved the women detecting those role related demands that were dispensable (“what you think you should do”) and accepting that they had to “sacrifice [some things] to achieve”. The women ensured they met essential role demands, and attempted to meet desirable role demands where possible. The women felt comfortable discarding dispensable role demands when faced with inadequate time, even if they had to “upset somebody.” Dispensable role demands were discarded passively by being “quite comfortable to not put my hand up for things I don’t think I’m either capable of doing or I don’t have the time to do” and more actively by being “really good at saying no.” Although the women often felt compelled to attend to all role demands, they accepted they needed to make priority choices to prevent their circumstances dictating which role demands were met.

Recognizing a hierarchy of role importance improved the efficiency of priority choices. A common guiding principal was “When it comes to juggling things, my priorities are that people are more important.” This translated to their role as a mother being perceived as the most important, followed by other family roles (such as parental carer and wife) and then working “within the constraints of work.” This hierarchical pattern was embedded into the women’s lives through deliberately curbing their career ambitions by “holding [themselves] back.” Although they expressed an element of “regret”, they accepted this was necessary in living consistently with their hierarchy of role importance. While this hierarchy of role importance was stable across family and work related roles, the relative importance of the domestic and self-maintenance roles varied between the women and over time. The women exhibiting higher between-role balance tended to prioritize activities addressing their health and wellbeing needs, whereas those who prioritized activities that met their domestic responsibilities reported lower between-role balance. Their personal hierarchies of role importance also facilitated between-role balance, allowing the women to attend to non-urgent demands within important roles, and ignore more urgent demands from less important roles.

Scheduling time and energy refers to the process of determining the commencement time and duration for each planned activity, including if the activity was to take place in isolation or combination. Scheduling was utilized to minimize the potential of “everything happening” at once, and resulted in “a fairly organized schedule”, allowing time to undertake time critical tasks and meet other prioritized role demands. Schedules were considered “tight” when dominated by numerous time critical tasks, such as their children’s extracurricular activities, parent’s specialist appointments and set work shifts. Once time critical tasks were scheduled, the women scheduled time for tasks from their lists of “what I want to achieve or get done”, with the amount of time budgeted based on estimates of “the correct amount of attention” required for each task. Given the complexity of their lifestyles, times when their multiple role demands “all fell into place” were revered, illustrated through “they all fitted in beautifully.” To fit all role demands into their schedule, the women used a combination of compartmentalizing roles, compatible role overlap, and continuously juggling roles.

Compartmentalizing roles, through scheduling one role at a time, was explained with the analogy that “Each little thing that you do is like a suitcase and you compartmentalize yourself in those roles… You can then just shut the other [roles] down as you move into the new role.” This strategy allowed the women to “make a decision that if I'm doing something with my family I'm going to be 100% there… You just accept it and enjoy it.” This decision to schedule a role on its own, and enforce boundaries, provided the women with the opportunity to focus their attention exclusively on their most important role at that time.

Compatible role overlap was favored when the women scheduled two or more roles simultaneously in a mutually beneficial way, and this was described with the metaphors “Killing more than one bird with one stone” and “A lot of my roles dovetail in together.” The women used compatible role overlap to “get it all done in that short space of time.” Examples included walking their dog with a friend, grocery shopping with their parents, going to a beachside café with their child and parent, or taking their children to drop-off an item for work.

Continuously juggling roles involved scheduling at least two roles at once, with an expectation that they would shift their attention between the roles as required. Examples included planning to supervise a child’s craft activity while cooking dinner or paying a parent’s bills while completing computer-based duties at work. Juggling could occur at a slower pace, where the women felt “You’ve got to try and snatch time wherever you can” by scheduling tasks (such as studying) in small gaps between other tasks. Juggling also occurred at a rapid pace where the women planned to oscillate between multiple tasks across numerous roles. The process of juggling involved constantly reassessing the relative priority of each role demand and continuously readjusting schedules. The juggling metaphor highlights that the process of achieving between-role balance required continuous effort and “an alert state” across their full range of roles.

A framework that balances structure with flexibility to manage time and energy refers to the women’s simultaneous integration of organization and spontaneity within their routines. This framework was utilized to assimilate the dichotomy of typical and unexpected role demands experienced within their lives. This strategy was dynamic and implemented by moving along a continuum from structure to flexibility.

Structure provided the women with confidence “that you can achieve a certain amount in every day” and there was agreement that “Organization is the main key” to balancing multiple roles. Some women relished being “very organized and…very meticulous”, illustrated through “I'm very systematic … to the point where it could be boring… to someone else, but I find it very exciting.” In contrast, remarks such as “It sounds really regimented and I don’t always like to lead my life like that” and “We all live such mundane lives” suggested that living a life dictated by a rigid structure was from some women out of necessity, rather than natural preference. Regardless of individual preferences, it was apparent that a degree of structure was necessary in achieving between-role balance.

Flexibility was also highly valued by the women, with many agreeing with the sentiments “Flexibility is the key to surviving” and “Spontaneity is important.” Flexibility occurred naturally in the women’s routines at the end of the work week, when “there’s not that pressure. It’s a mental thing. I’ve still got to get up the next day and do other things, but it’s just knowing you don’t have to go to your workplace.” School holidays also provided “a lot more free time, because you don’t have to worry about lunches, school bags, folding up uniforms.” Flexibility supported the women in budgeting their time and energy. Flexibility was described as the most effective approach in managing change, illustrated by “Kids are ever-changing… The more you try to be rigid, the more tears there are… I guess flexible is a good word—but I think it’s unorganized, but never mind. It seems to all work in the end.” Recognizing the importance of flexibility also supported the women to overcome feelings of disorganization, which resulted from a more reactionary approach to meeting role demands. An example was “There aren’t many days I … get to do the stuff on my list in order. It changes all the time… The biggest thing I’ve learnt is it’s okay to not be that organized. It’s organized, but it’s organized chaos.” Hence, a degree of flexibility was necessary to achieve between-role balance.

Balancing structure and flexibility enabled the women to meet both essential and desirable role demands, and buffered the impact of unexpected events or delays. Adopting a more structured approach during the week allowed the women to focus on essential role demands and created opportunities for the women to focus on more desirable role demands on the weekend. This was explained with:

Sometimes I allocate times for cooking. I hate doing that. It’s not really how I want to live my life, but it’s just to get everything done…. There was a time I would go out more in the evenings, whereas now I tend to think that I enjoy the weekends so much more if I spend that time at home… I can pay the bills and maybe prepare a few meals in those evenings that I'm in, so that releases time at the weekends.

Being proactive in managing tasks, such as maintenance or being prepared, was used to “bank time” and provided a buffer against unexpected events or delays. This approach allowed the women to “set up for a bit more success”, illustrated with: “If home maintenance is relatively up to date then you’re better able to let it drop to the bottom of the priority list.” In the short term the women advocated “planning ahead for the next day, and thinking about whatever [they could] get done in a day to make the next day better.” Scheduling unallocated time, either on a daily or weekly basis, provided the women with the opportunity to engage in spontaneous activities, renew energy and manage unanticipated challenges.

#### Releasing responsibility–“You’re not the only one that can do things”

Releasing responsibility to others refers to the women’s strategy of sharing selected tasks with others. This strategy was utilized when 17 women felt they had more role responsibilities than they were capable of meeting. The life lesson that “You’re not the only one that can do things” was often discovered after many years of where they felt the need to “control everything.” As a result the women learnt to become comfortable with playing a project management role, by delegating, promoting independence or outsourcing, in order to free up their time and energy.

Delegating tasks was enabled by overcoming the reluctance to ask for assistance with responsibilities, rather than waiting for support to be offered. One woman had previously harboured anger and resentment that her husband did not offer assistance, however she had since:

Realised what an injustice I had done him, because I never, ever had given him the opportunity to step up to the plate, because I just did it. Why didn't he see that I needed [help]? And then my counsellor said to me, “But you didn't ask either”.

This example highlights how delegating broke the vicious cycle of “while you’re willing to just keep giving, everyone’s willing to keep taking.” The women described learning to disengage from responsibilities once they were delegated and accept the potential decreases in quality or frequency.

Promoting independence among other people within their social network was an indirect approach to freeing up time and energy. This involved encouraging others to be more independent through “teaching them and releasing responsibility.” Strategies included involving children in domestic tasks from a very young age, not rescuing older children if they had neglected to complete a task, expecting their spouse to participate in childcare, refusing to assist parents with chores when they were eligible for formal services and accepting when colleagues completed projects on their behalf.

Outsourcing tasks to paid services allowed the women to dedicate their scarce resources on the most valued aspects of their roles. Several of the women hired a cleaner or gardener at home and those with younger children typically utilized childcare facilities. When eligibility criteria was met, the women encouraged their parents to access support services from community agencies, including those that assisted with cleaning, gardening, transport or social activities. One woman planned to hire a bookkeeper “to keep my pile down … and keep up to date” within her home based business. Collectively, outsourcing tasks across a number of roles allowed the women to enhance their between-role balance.

#### Nurturing social connection–“They need a little maintenance”

Nurturing social connection refers to the women’s strategy of fostering individual relationships or developing communities characterized by a culture of helping others. This strategy was utilized by 17 women to create a life “full of love hearts and kisses”, secure ongoing social support and experience the psychological benefits of caring for others. In addition to displaying affection to loved ones through “nurturing, hugging, acknowledging, and being interested in them”, the women nurtured social connections through realizing the importance of relationships and prioritizing social activities in their schedule.

Realizing the importance of relationships with loved ones helped the women to prioritize nurturing these connections, especially in relation to their spouse and friends. The connection with their spouse was nurtured because the “role as a wife is the one that’s probably going to sustain all the other relationships… the one that hopefully will last forever. And it’s the one that we started with, so it’s good to work on, it’s important.” The women with older children were particularly aware of the need to focus on their marriage, with one woman stating “we need to nurture each other now, it’s our turn now to start really looking after one another… We have to make sure that our relationship stays really strong now to be able to look after one another in our ripe old age.” The comment that “friends are precious and are like gardens—they need a little maintenance” demonstrated that the women also recognized the importance of nurturing connection with friends.

Prioritizing social activities in their schedule was achieved through placing family and friend roles in a higher position in their hierarchy of role importance, and operationalizing this through committing to frequent social activities together. Regular social activities with their partner included going on a lunch date, dining out with other couples, entertaining at home, playing sport, working together or time spent together at home talking, watching television, cuddling or “intimate moments … if he’s lucky.” The women prioritized “chatting” with friends, and this was typically combined with an activity such as having a manicure, attending a book club, exercising or a play date with children. These friendships were resilient and lasting, despite infrequent opportunities to meet:

The kinds of friends that I do have are very close friends… If you have not seen them for several months, you take off just like it was yesterday, because they’re old friends and they know and understand you. And I think that’s a wonderful thing… They are very important relationships that I’ve maintained and would never compromise.

The women in this study prioritized social activities that developed a family based community focused on nurturing intergenerational relationships between their children and parents: “I try really hard to take the boys [to my father’s house] at least once a week.” The women also developed wider communities through regular attendance at their children’s school and sports events.

#### Reciprocating–“There’s kind of this pay-off”

Reciprocating refers to the women’s strategy of establishing mutually beneficial relationships, where “there’s kind of this pay-off” and neither party felt guilty asking for help or burdened providing help. This strategy was utilized by 15 women to “share the load” in meeting their multiple role demands, through exchanging practical support (such as assistance with childcare, domestic tasks and transport) or emotional support (such as companionship and “somebody to talk to”). Reciprocating built on the connections they had nurtured with individuals and communities, and was enhanced through recognizing the continuity, lack of linearity, and complexity associated with their reciprocal relationships.

Continuit*y* was fostered within reciprocal relationships as much as possible, despite the frequently observed “role reversal” that occurred in their relationships with their parents. An example of this consistency in support was “he’s still my dad and I still learn from him … sometimes he just says things and I go ‘ah’. He puts it all in perspective, and he’s always been that for me.” By finding ways to maintain patterns of support, the women contributed to their own, and their loved ones’, between-role balance.

A lack of linearity was associated with reciprocating, because “life works in a way that you don’t necessarily get direct help from the person that you’ve helped… It may be someone else that’s giving you help and you’re helping someone else, but it all irons out in the long run.” This understanding encouraged the women to participate in a culture of helping others, and the reciprocal benefits aiding their between-role balance.

Complexit*y* occurred within reciprocal relationships by intertwining roles, as explained with “it’s important that I'm … giving my best to my husband…. The most valuable thing I’ve ever given someone are children…. And through that I feel a responsibility… to give his children the best of me.” Hence, reciprocating needed to take into account this complex and interconnected nature to effectively facilitated between-role balance.

## Discussion

The range of individual role balance strategies that the working ‘sandwich’ generation women from this study utilized supported previous observations that managing multiple roles is complex, multidimensional and interconnected [6,7,29,49,56], and reinforce the finding that conscientious effort is required to overcome these challenges. Just as the working mothers studied by Wada and colleagues [49] utilized conflicting approaches to balance their roles, the women in this study utilized opposing strategies simultaneously to achieve and maintain role balance. Examples included balancing the dichotomies of “being the best you can” with “repressing perfectionism” and “promoting independence” with “nurturing social connections.” The Model of Juggling Occupations [12] provided an appropriate conceptual framework to explore these complex individual role balance strategies in a holistic and detailed manner, even though the final strategies identified did not correspond directly to the original concepts because of their overlapping and interconnected nature.

The most important individual role balance strategy emerged as “maintaining health and wellbeing.” Although this strategy is implemented within the self-maintainer role, it is considered a between-role strategy due to its many benefits across the entire role system. The holistic range of self-care activities and maintenance strategies described, along with suggestions for optimizing motivational factors and routines to overcome barriers to implementation, expands greatly on previous research findings [6,8,47,54–56]. Activities and maintenance strategies within the physical health domain revolved around engaging in healthy habits to optimize current and long-term outcomes [6,47,54,55]. Activities and maintenance strategies within the mental health domain were focused on making and protecting time to reflect alone or with others, thus overcoming the challenge of finding time for themselves [8,55]. Finally, activities and maintenance strategies within the wellbeing domain were focused on pursuing leisure [6,8,9,54–56]. Although “maintaining health and wellbeing” was a strategy utilized by most of the women in this study on a regular basis, other studies of this particular sample (upcoming publications) demonstrated they experience time inadequacy in relation to their self-maintainer role and spent less time participating in sleep, personal care and leisure compared to other working mothers. It is possible that these women consider attempting to “maintain health and wellbeing” as synonymous with achieving role balance. Thus, they may have been more likely to volunteer for this study if they successfully integrated this strategy into their lifestyle, and hence felt they would be better able to contribute valuable information. Although this self-selection bias may reduce the transferability of the findings to the working sandwich generation population in general [67], this expertise was also beneficial to this study, where the expressed aim was to obtain insights on their role balance strategies.

Other within-role and between-role balance strategies identified in this study extend knowledge regarding the individual role balance strategies described in the ‘sandwich’ generation [6–9,22,33,34,36,74] and occupational therapy literature [18,49–60]. The within-role balance strategies identified in this study expand on Neal and Hammer’s [7] strategies of decreasing cognitive demands by prioritizing activities that are important, enjoyable or achievement focused, and decreasing emotional demands by having a greater awareness of values, interests and skills. Whilst some between-role balance strategies corresponded directly to Neal and Hammer’s model [7], such as “repressing perfectionism” and “decreasing expectations of self”, other strategies overlapped with several of their approaches (for example “managing time and energy” overlapped with “prioritizing activities” and “planning”). In addition, the between-role and within-role balance strategies used by the women in this study are aligned with popular approaches to improve mindfulness [75], personal effectiveness [76], flourishing [77] and whole-hearted living [78].

Despite previous research linking financial strain and coping strategies [17], socio-economic factors did not impact substantially on the use of individual role balance strategies within this study. There are several possible explanations for these unexpected finding. The women studied were relatively affluent and may not have experienced financial strain or economic barriers to implementing their chosen individual role balance strategies. This financial security is likely to explain in part why the vast majority of these women worked part-time hours, a circumstance that allowed greater access to the valuable commodity of time. In addition, the majority of individual role balance strategies identified in this study had no associated financial costs, as they relied on activity choices, cognitive processes and social negotiations. The only exceptions were selected strategies for meeting health or wellbeing needs (e.g., health screenings or treatments, formal exercise programs, counseling and pampering) or outsourcing tasks to paid services (e.g., cleaner, gardener, childcare facility and aged care service). In the Australian context where this study occurred, many of these expenses would have been subsidized or fully sponsored through government funded systems for health, childcare and aged care [79–81]. In addition to private health insurance schemes and corporate funded wellness programs, Australia has a comprehensive government funded health care system to ensure that physical and mental health treatments are within the reach of all citizens [79]. Childcare fees are subsidized for all working parents in Australia, with higher benefits paid to lower income families [80]. The Australian aged care system provides extensive assistance to allow the parental generation to live independently in their own home, with services including personal care, domestic help, meals, transport, social activities, health interventions, equipment or home modifications and respite care [81]. Although a related study of the same sample of 18 women did not find any quantitative evidence that any socio-demographic factors or many role related characteristics were associated with role balance (upcoming publication), it is possible that a survey of a much larger sample of working ‘sandwich’ generation women would have the statistical power to identify socio-demographic factors and role related characteristics or stages that predict the use of individual role balance strategies and role balance outcomes [67].

Recommendations for health professionals include the establishment of group based lifestyle approach intervention programs for working ‘sandwich’ generation women, to assist in the development of a toolkit of individual role balance strategies [13,33,54]. These intervention programs would allow women to build a community with other working ‘sandwich’ generation women [4]. Participation rates could be enhanced through scheduling intervention programs at workplaces [74] or with concurrent activities for their parents and children to address limitations in availability because of their multiple family responsibilities [9]. In addition to drawing on the findings from this study, intervention programs could also incorporate existing evidence based approaches, such as acceptance commitment therapy [75] and positive psychology [77]. It is recommended that intervention programs include a mechanism for further data collection on individual role balance strategies, through an exploration of existing strategies at the program commencement and an evaluation of the effectiveness of strategies in improving role balance outcomes. It is anticipated that embedding research within an intervention program would provide access to working sandwich generation women with lower levels of role balance, as participants may perceive greater benefits and relevance associated with their involvement.

### Limitations

Given the paucity of research on the topic of individual role balance strategies among working ‘sandwich’ generation women, the intent of this study was to obtain an understanding of the depth and breadth of approaches utilized by these women. Consequently, there are a number of limitations of this study in terms of the research design and sample that provide opportunities for future research.

In terms of the research design, reliance on a single interview to identify individual role balance strategies limited our understanding, hence longitudinal or multiple method studies may offer further insights [49,57,82]. A single interview also restricted investigations into the frequency, consistency or extent that each woman utilized the 12 types of individual role balance strategies identified. Future research exploring the relationship between utilization of role balance strategies and role balance outcomes would be beneficial to provide an evidence base for future intervention programs [67]. This study is limited in relation to our understanding of the relative importance of the individual role balance strategies employed by working ‘sandwich’ generation women. Future research into the varied viewpoints on the relative importance of these role balance strategies would be beneficial. Finally, although the women were all partnered, it is likely that the women received varying levels and types of support from their spouse. It is recommended that future research studies collect information on the type and amount of emotional and practical support provided by partners, to better understand how this environmental factor mediates the effectiveness of individual role balance strategies.

In terms of the sample, whilst a convenience sampling approach was selected as the most practical option, the resulting self-selection bias may have influenced the findings [66,67]. The women who volunteered to participate in this study had greater access to economic resources, spousal support, community facilities and flexible work and care arrangements, compared to the overall population, hence the individual role balance strategies employed may differ to other working ‘sandwich’ generation women with less supports. Future research should focus on studying a broader range of working ‘sandwich’ generation women, including a greater variation in socio-economic background, marital status, residential location (such as rural or remote), employment conditions and parental care arrangements (for example parents living in residential care). This more representative sample may be recruited from future large-scale surveys of women if demographic questions were included for all three roles of mother, parental carer and worker. Finally, this study was restricted to women and it is anticipated that men combining the roles of father, parental carer and worker would experience different role related challenges, individual role balance strategies and role balance outcomes to their female counterparts. Future research should subsequently explore the individual role balance strategies utilized by working sandwich generation men.

## Supporting Information

S1 File Interview Guide. (PDF)
