# Supplementary material for: Working Sandwich Generation Women Utilize Strategies within and between Roles to Achieve Role Balance
Source: PLoS One. 2016 Jun 15;11(6):e0157469. doi: 10.1371/journal.pone.0157469 (PMC4909236; doi:10.1371/journal.pone.0157469)
Supplement: S1 File — (PDF) [file pone.0157469.s001.pdf]

S1 File. Interview Guide

1. During the first section of the interview I would like for us to talk about "role balance" in the context of *your* life.

a. Can you tell me about what the phrase "role balance" means to you?
b. How satisfied are you with your current role balance?
c. What factors in your life help you to achieve role balance?
d. What factors in your life get in the way of achieving role balance?
e. How does your current role balance impact on your health and wellbeing?
f. How does your current role balance impact on your different roles in life?

2. The following section of the interview focuses on your role as a *worker*.

a. What does this role involve?
b. What are the best, or most enjoyable, aspects of this role?
c. What are the worst, or least enjoyable, aspects of this role?
d. How do your habits or routines within this role impact on your role balance?
e. Are you satisfied with these habits and routines?
f. What are the most important or valuable aspects of this role for you?
g. How often do you experience these important or valuable aspects?
h. How does this impact on your role balance?
i. What things within this role do you feel you do well, or are proud of?
j. What is the biggest challenge you are currently facing within this role?
k. How successful do you think you will be within this role over the next six months?
l. How do you feel your level of confidence within this role impacts on your role balance?

3. The following section of the interview focuses on your role as a *mother*.

a. What does this role involve?

b. What are the best, or most enjoyable, aspects of this role?

c. What are the worst, or least enjoyable, aspects of this role?

d. How do your habits or routines within this role impact on your role balance?

e. Are you satisfied with these habits and routines?

f. What are the most important or valuable aspects of this role for you?

g. How often do you experience these important or valuable aspects?

h. How does this impact on your role balance?

i. What things within this role do you feel you do well, or are proud of?

j. What is the biggest challenge you are currently facing within this role?

k. How successful do you think you will be within this role over the next six months?

l. How do you feel your level of confidence within this role impacts on your role balance?

4. The following section of the interview focuses on your role as a *parental care giver*.

a. What does this role involve?

b. What are the best, or most enjoyable, aspects of this role?

c. What are the worst, or least enjoyable, aspects of this role?

d. How do your habits or routines within this role impact on your role balance?

e. Are you satisfied with these habits and routines?

f. What are the most important or valuable aspects of this role for you?

g. How often do you experience these important or valuable aspects?

h. How does this impact on your role balance?

i. What things within this role do you feel you do well, or are proud of?

j. What is the biggest challenge you are currently facing within this role?

k. How successful do you think you will be within this role over the next six months?

l. How do you feel your level of confidence within this role impacts on your role balance?

5. This section looks at your experiences of being a working mother and having caring responsibilities for your aging parent(-in-law).

a. What have been the positive aspects of combining these roles?

b. What have been the negative aspects of combining these roles?

c. How has having the combination of these three roles impacted on your other roles in life?

d. How satisfied are you with your level of participation in these other roles?

6. The final section of the interview allows you to make any closing comments or discuss any other ideas in more detail.

a. What are the main characteristics of a day where you feel a high level of role balance?

b. What are the main characteristics of a day where you feel a low level of role balance?

c. What advice would you give to other working women caring for their parents or parent-in-laws and raising children, to help achieve balance between different roles?

d. Is there anything else you would like to mention or share as part of this interview?
